# Cytolethal distending toxin enhances *Escherichia coli* urinary tract infection

**DOI:** 10.1128/iai.00103-26

**Published:** 2026-07-13

**Authors:** Santosh Paudel, Benjamin E. Curtis, Siena Stefa, Harry L. T. Mobley, Melanie M. Pearson, Mark T. Anderson

**Affiliations:** 1 Department of Microbiology and Immunology, University of Michigan, Michigan Medicine 242912, Ann Arbor, Michigan, USA; 2 Unit for Laboratory Animal Medicine Pathology Core, University of Michigan, Michigan Medicine 21614, Ann Arbor, Michigan, USA; St Jude Children's Research Hospital, Memphis, Tennessee, USA

**Keywords:** urinary tract infection (UTI), genotoxin, uropathogenic *E. coli *(UPEC), cytolethal distending toxin (CDT)

## Abstract

Cytolethal distending toxin (CDT) is a virulence factor produced by several gram-negative bacteria, including *Escherichia coli*, the most prevalent etiological agent of urinary tract infections (UTIs). CDT causes DNA damage to eukaryotic cells, leading to the impairment of host defenses by disrupting epithelial barriers, suppressing acquired immunity, and promoting pro-inflammatory responses. *E. coli* strains encoding CDT have been previously identified in samples from UTI patients; however, the specific function of CDT in the development of UTI remains undefined. In this study, we used a mouse model of ascending UTI to determine the role of CDT during infection. An *E. coli* mutant strain lacking the *cdtABC* locus was generated and combined with wild-type bacteria to co-infect mice via transurethral inoculation. At 1 day post-inoculation, competitive indices demonstrated a significant disadvantage for the *cdt* mutant in urine, bladder, and kidneys. Single-strain infections were also performed as a further assessment of CDT impact, demonstrating that the *cdt* mutant had reduced kidney colonization, indicative of CDT contributions to pyelonephritis. Histopathological analysis of the urinary bladder and kidney tissues from mice infected with CDT-encoding *E. coli* demonstrated higher levels of inflammation and tissue damage within the kidneys at both 1 and 7 days post-inoculation and in the bladder after 7 days when compared to mice infected with *cdt* mutant bacteria. Collectively, these findings identify a role for CDT in UTI pathogenesis.

## INTRODUCTION

Cytolethal distending toxin (CDT) is a bacterial genotoxin secreted by several pathogenic gram-negative bacteria including *Aggregatibacter actinomycetemcomitans* (1), *Campylobacter jejuni* (2)*, Escherichia coli* (3), *Haemophilus ducreyi* (4), and *Salmonella* spp (5, 6). In *E. coli*, five variants of CDT (CDT-I through CDT-V) have been recognized and are differentiated by their genomic location (7, 8). CDT-II is chromosomally encoded (9), CDT-III is located on a pVir plasmid (10), CDT-I and CDT-IV are associated with lambdoid prophage (11), and CDT-V can be found within chromosomal genes flanked by bacteriophage P2 and lambda-like sequences or in inducible bacteriophages (12). All variants have been observed in pathogenic *E. coli* strains of both intestinal and extraintestinal origin (3, 13, 14).

CDT is most often observed as a heterotrimeric protein complex composed of the subunits CdtA, CdtB, and CdtC. Each of the five recognized *E. coli* CDT variants includes all three proteins. The components of types I and IV have high amino acid sequence identity (≥85%) to each other, whereas the components of types II, III, and V have similarly high identity to each other but lower identity to types I and IV (8). The CdtA and CdtC subunits facilitate host cell binding and cellular internalization of the CdtB subunit via interaction with cholesterol-rich membrane domains (15), and in some species, through binding of CDT-containing extracellular vesicles to host cell glycans (16). CdtB is the catalytically active subunit of the toxin, possessing DNase (17) and phosphatase activity (18). CdtB DNase activity induces double-strand DNA breaks in mammalian cells, triggering host DNA damage response pathways and leading to cell cycle arrest, apoptosis, and inflammation (17). The phosphatase activity is less well characterized and is thought to support toxin entry and nuclear trafficking (18, 19).

*E. coli* CDT induces characteristic cytopathic effects across several epithelial cell types (e.g., HeLa, U2OS, RKO, Hep-2, and HCECs), including megalocytosis, chromatin fragmentation, and multinucleation (3, 20, 21). These phenotypes stem largely from G2/M cell cycle arrest and defective DNA repair, resulting in genomic instability and pro-tumorigenic signaling (22, 23). Although CDT-mediated cytotoxicity has been extensively described in intestinal and colonic epithelial models (20, 24, 25), including CDT-positive *E. coli* isolated from the gastrointestinal tract (3, 20), the contribution of CDT to urinary tract infection (UTI) remains undefined.

UTIs represent a significant global health burden which has become increasingly prevalent over the past 30 years (26). Uropathogenic *E. coli* (UPEC) accounts for about 75% of uncomplicated, and 65% of the complicated UTI cases (27). UPEC employ a diverse array of virulence factors that contribute to adherence, colonization, immune evasion, and tissue damage within the urinary tract (28–31). CDT-producing *E. coli* has been identified among UTI isolates at a rate of approximately 10% in limited sample sizes (14, 32, 33), but its role in the urinary tract has not been defined. This study investigates the contribution of CDT to UPEC virulence during UTI by assessing bacterial burden, inflammatory responses, and tissue pathology in the bladder and kidneys in an effort to advance our understanding of factors that drive these infections.

## RESULTS

### UPEC *cdt* genes are expressed in urine and stimulate DNA damage responses

We identified putative *cdt* genes via homology search from the previously reported genome sequences of two uncomplicated *E. coli* UTI isolates, HM56 and HM57 (32) (Table 1). CDT subtype was inferred by aligning published CDT-typing primer sequences to the HM56 and HM57 genome assemblies. The CDT-IV specific primers matched the *cdt* operon of HM56, while the CDT-I specific primers matched the *cdt* operon of HM57, consistent with classification as CDT-IV and CDT-I, respectively (14). Both CDT-I and CDT-IV have been reported to be carried within lambdoid prophage regions in the *E. coli* genome (11). The PHASTEST platform (34) was used to confirm prophage locations and annotate each of the intact prophage regions in HM56 (*n* = 4 regions) and HM57 (*n* = 4 regions). The *cdt* genes were encoded in region 4 for HM56 and region 3 for HM57 (Fig. S1).

**TABLE 1:** Bacterial strains and plasmids used in this study

Name	Genotype or description	Source
Bacteria		
*E. coli* HM56	Wild-type	(32)
*E. coli* HM56	Δ*cdt*::*kan^R^*	This study
*E. coli* HM57	Wild-type	(32)
*E. coli* HM57	Δ*cdt*::*kan^R^*	This study
Plasmid		
pKD4	Source of FRT-*kan^R^*-FRT marker	(35)
pSIM18	Encodes λ red recombineering system, hygromycin^R^	(36)
pBBR1MCS-4	Cloning and shuttle plasmid vector	(37)
pBB-*cdtABC*	pBBR1MCS-4 containing HM56 *cdtABC*	this study

**Fig 1:**
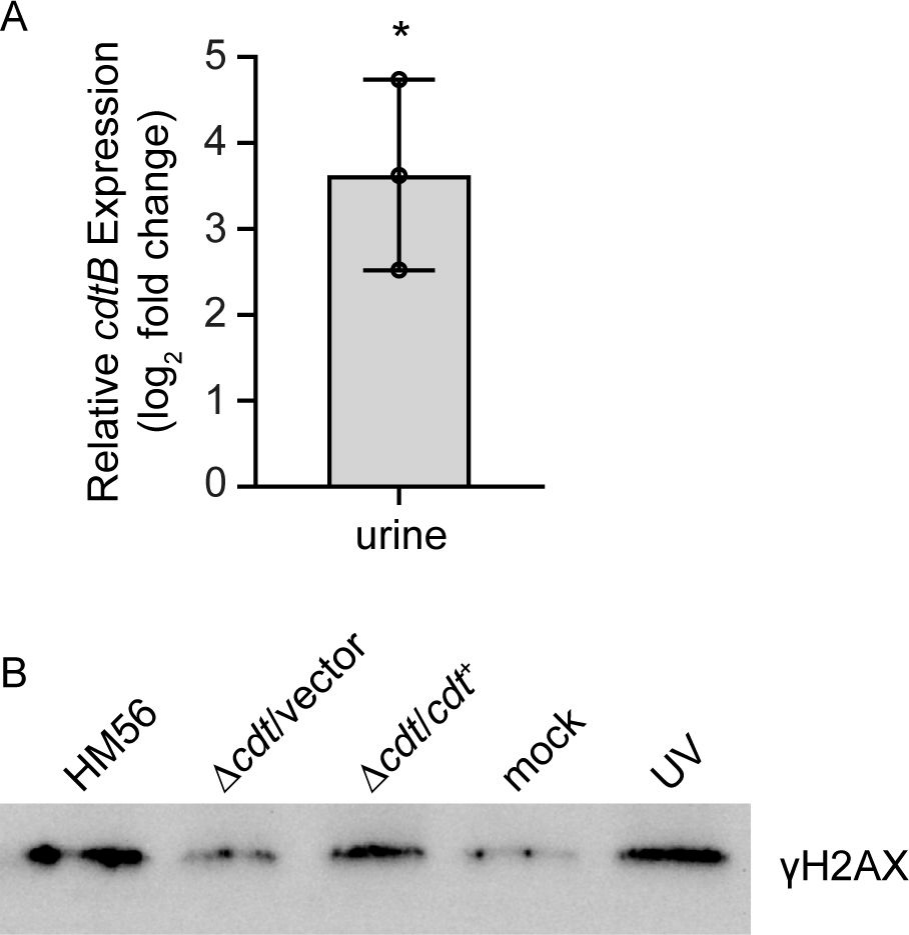
Expression of *cdtB* in human urine and DNA damage response of bladder epithelial cells co-cultured with HM56. (**A**) *E. coli* HM56 was exposed to human urine or LB medium for 1 h, followed by quantitation of *cdtB* transcript levels by quantitative PCR. The mean (±standard deviation) *cdtB* expression from three biological replicates in urine relative to LB is shown. Statistical significance was assessed by one-sample *t*-test and a theoretical mean of zero: *, *P* < 0.05. (**B**) T24 bladder epithelial cells were inoculated with wild-type HM56, the Δ*cdt* mutant harboring vector control plasmid pBBR1MCS-4, or the complemented mutant with *cdtABC* genes provided in *trans* and co-cultured for 5 h. An immunoblot for phosphorylated H2AX (γH2AX) was performed on total cell lysates with mock-treated cells and cells exposed to ultraviolet (UV) light serving as negative and positive controls for DNA damage, respectively.

To determine whether *cdt* genes are expressed under conditions that are relevant to UTIs, we measured the abundance of HM56 *cdtB* transcripts in human urine. Bacteria that were exposed to urine for 1 h had approximately 12-fold higher *cdtB* expression compared to bacteria in standard lysogeny broth (Fig. 1A). Therefore, *cdtB* is actively transcribed in urine, and these results suggest that the *cdt* locus may also be expressed under similar conditions in the urinary tract.

CDTs initiate host cell DNA damage due, in part, to their DNase activity. To test the role of UPEC CDT in genotoxicity, bacteria were co-cultured with T24 bladder carcinoma cells, followed by immunoblot of phosphorylated histone subunit H2AX (γH2AX) as a marker of DNA damage responses. Wild-type HM56 bacteria elicited detectable γH2AX signal 5 h after inoculation, similar to the response of T24 cells exposed to an ultraviolet light source (Fig. 1B). A mutant derivative lacking the *cdtABC* genes (Δ*cdt*) showed diminished γH2AX reactivity that was partially restored by genetic complementation with plasmid-borne *cdtABC* genes, as assessed by densitometry normalized to cellular proteins (Fig. S2). CDT therefore contributes to the genotoxicity of bladder epithelial cells in culture.

### CDT confers a fitness advantage in the urinary tract

**Fig 2:**
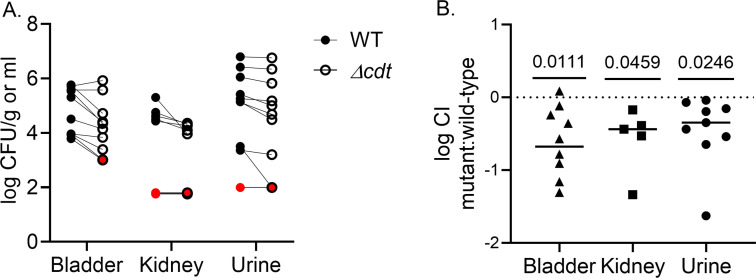
CDT contributes to UTI fitness of *E. coli*. CBA/J mice (*N* = 10) were transurethrally inoculated with equal numbers of wild-type (WT) HM56 and Δ*cdt* mutant HM56. Urine, bladders, and kidneys were collected for CFU enumeration at 24 h post-inoculation. (**A**) CFU of each strain recovered from individual mice. Red symbols indicate no CFU recovered after plating. (**B**) CI of the ∆*cdt* mutant compared to wild-type. Statistical significance was assessed by a one-sample *t*-test, comparing the mean log CI (lines) to a hypothetical value of zero for neutral fitness (dotted line): Statistically significant if *P*-values < 0.05.

To initially assess the fitness contribution of CDT during UTI, we performed co-challenge infections with wild-type HM56 and the Δ*cdt* mutant (1:1) in a murine model. Urine, bladder, and kidneys were recovered from infected mice followed by the quantitation of wild-type and mutant bacteria at each site (Fig. 2A). The competitive index (CI) indicates a significant fitness disadvantage for ∆*cdt* mutant bacteria throughout the urinary tract at 24 h post-inoculation (Fig. 2B). Relative fitness of the mutant was lowest in the bladder, with an 8-fold difference compared to wild-type bacteria.

**Fig 3:**
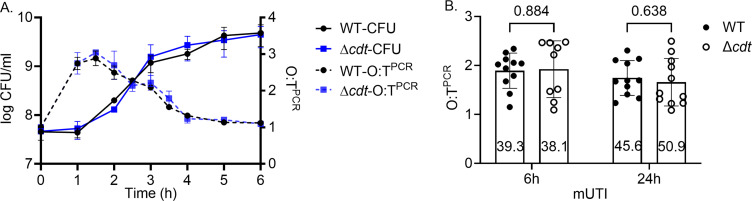
Wild-type HM56 and the ∆*cdt* mutant have similar growth kinetics in culture and during infection. (**A**) CFU from wild-type (WT) and Δ*cdt* cultured in LB are plotted as the mean (*N* = 2) ± the standard deviation or as O:T^PCR^ (*n* = 9–11). (**B**) O:T^PCR^ values from the urine of infected mice collected at 6 or 24 h are plotted with inset numbers indicating the doubling time derived from the median O:T^PCR^ (bars). Statistical significance was assessed using the Mann–Whitney U test. *P*-values are displayed on the graph, and neither was below the 0.05 significance threshold.

To determine whether the UTI fitness defect of the Δ*cdt* mutant was attributable to differences in bacterial growth rate, we compared the growth kinetics of the HM56 wild-type and Δ*cdt* mutant strains under *in vitro* and *in vivo* conditions. No significant difference in the population doubling time was observed between these strains in lysogeny broth (LB) medium calculated from the CFU measured during the exponential growth phase from 1 to 3 h post-inoculation, calculated at 25.5 ± 1.2 and 25.7 ± 3.4 min, respectively (Fig. 3A). Similar experiments were also performed in human urine and RPMI medium, with minimal differences observed between the two strains in these conditions (Fig. S3). In preparation for measuring replication *in situ*, we also determined the growth rates of bacteria in LB culture by our previously reported O:T^PCR^ method (38). O:T^PCR^ measurements, a proxy for the relative abundance of genome origin and terminus copies in the population, were consistent across both strains and showed similar replication dynamics over 6 h (Fig. 3A). As expected, doubling times calculated from the highest O:T^PCR^ values (at t = 1.5 h) were also consistent at 19.7 ± 1.9 and 18.1 ± 0.3 min for wild-type and the Δ*cdt* mutant strains, respectively. Using the O:T^PCR^ method, we then investigated the growth rates of bacterial populations recovered from the urine of infected mice, and no significant difference was found in the doubling times of wild-type and the Δ*cdt* mutant at 6 or 24 h (Fig. 3B). Growth was fastest for both strains 6 h after inoculation (39.3 and 38.1 min doubling time) and slowed by 24 h (45.6 and 50.9 min doubling time) (Fig. 3B). Together, these results demonstrate that there was no inherent growth limitation of the ∆*cdt* mutant under the conditions tested and that the fitness defect of this strain is not attributable to differences in bacterial replication.

### HM56 CDT contributes to kidney colonization

**Fig 4:**
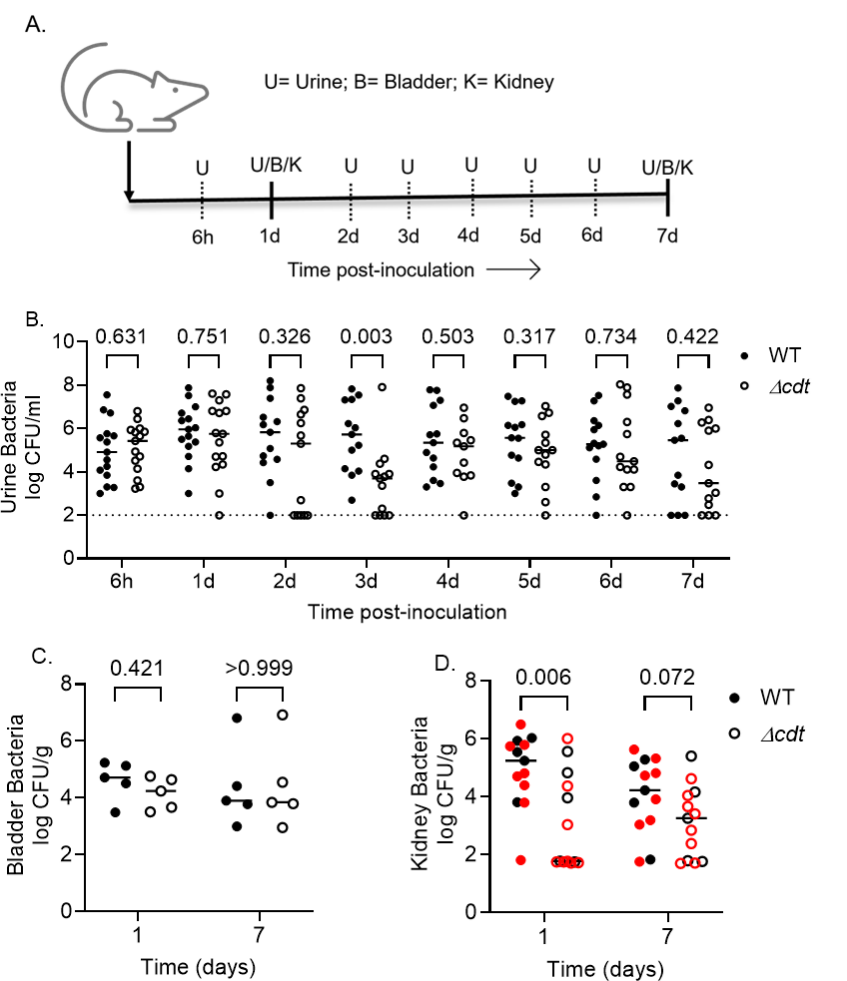
CDT contributes to short- and long-term kidney colonization in a single-strain UTI model. (**A**) Timeline of infection and bacterial enumeration in female CBA/J mice transurethrally inoculated with wild-type (WT) HM56 and Δ*cdt* mutant strains. (**B–D**) Bacterial burdens from individual mice in urine (**B**), bladder (**C**), and kidneys (**D**) (*n* = 5–15) with the median indicated by solid lines and the limit of detection (urine) indicated by the dotted line. The red symbols in panel D designate mice in which bacterial counts were derived from only one kidney; the remaining kidney was preserved for histology. Statistical significance was assessed using the Mann–Whitney U test. *P*-values are displayed in the graph, and values < 0.05 were considered statistically significant.

We further examined the role of CDT in colonization of the urinary tract using a single-strain infection model of ascending UTI, tracking bacterial abundance in the urine, bladder, and kidneys over the course of a week (Fig. 4A). Both wild-type and ∆*cdt* mutant bacteria persisted in the urine of mice throughout the 7-day infection period. However, urine bacterial burdens were highly variable, and there was no significant difference in CFU levels between the two strains at most time points (Fig. 4B). The one exception was observed at 3 days post-inoculation, where wild-type bacteria exhibited an approximately 2-log higher median CFU compared to Δ*cdt* bacteria (Fig. 4B). Both strains individually were similarly able to colonize the bladder, and no significant difference was observed between wild-type HM56 and ∆*cdt* mutant CFU at 1 or 7 days post-inoculation (Fig. 4C). In the kidney, wild-type bacteria exhibited a > 3 log higher CFU burden compared to the ∆*cdt* mutant at 1 day after inoculation, representing a significant increase in either colonization or bacterial survival at this site (Fig. 4D). Overall, bacteria were also recovered from a higher proportion of kidneys from mice inoculated with wild-type bacteria compared to those inoculated with the Δ*cdt* mutant after 1 day (92% and 46%, *n* = 13, *P* = 0.0302 Fisher’s exact test). A trend toward decreased ∆*cdt* kidney CFU (Fig. 4D) was also observed after 7 days; however, this difference was not significant. Together, these results demonstrate that CDT enhances kidney colonization by *E. coli*.

To determine whether CDT contributed to UTI in strain HM57, a Δ*cdt* mutant derivative was constructed and used to infect mice in comparison to wild-type bacteria. Both HM56 and HM57 wild-type control strains had similar day 1 bladder and kidney colonization levels (Fig. 4; Fig. S4). However, no significant difference in bacterial colonization was observed in the urine, bladders, or kidneys of mice inoculated with the HM57 Δ*cdt* mutant compared to wild-type bacteria (Fig. S4). The observed difference in results between HM56 and HM57 suggests that the contribution of CDT to UTI may be influenced by other variable UPEC factors or the specific CDT type encoded by each strain.

### CDT induces inflammation in the bladder and kidney

**Fig 5:**
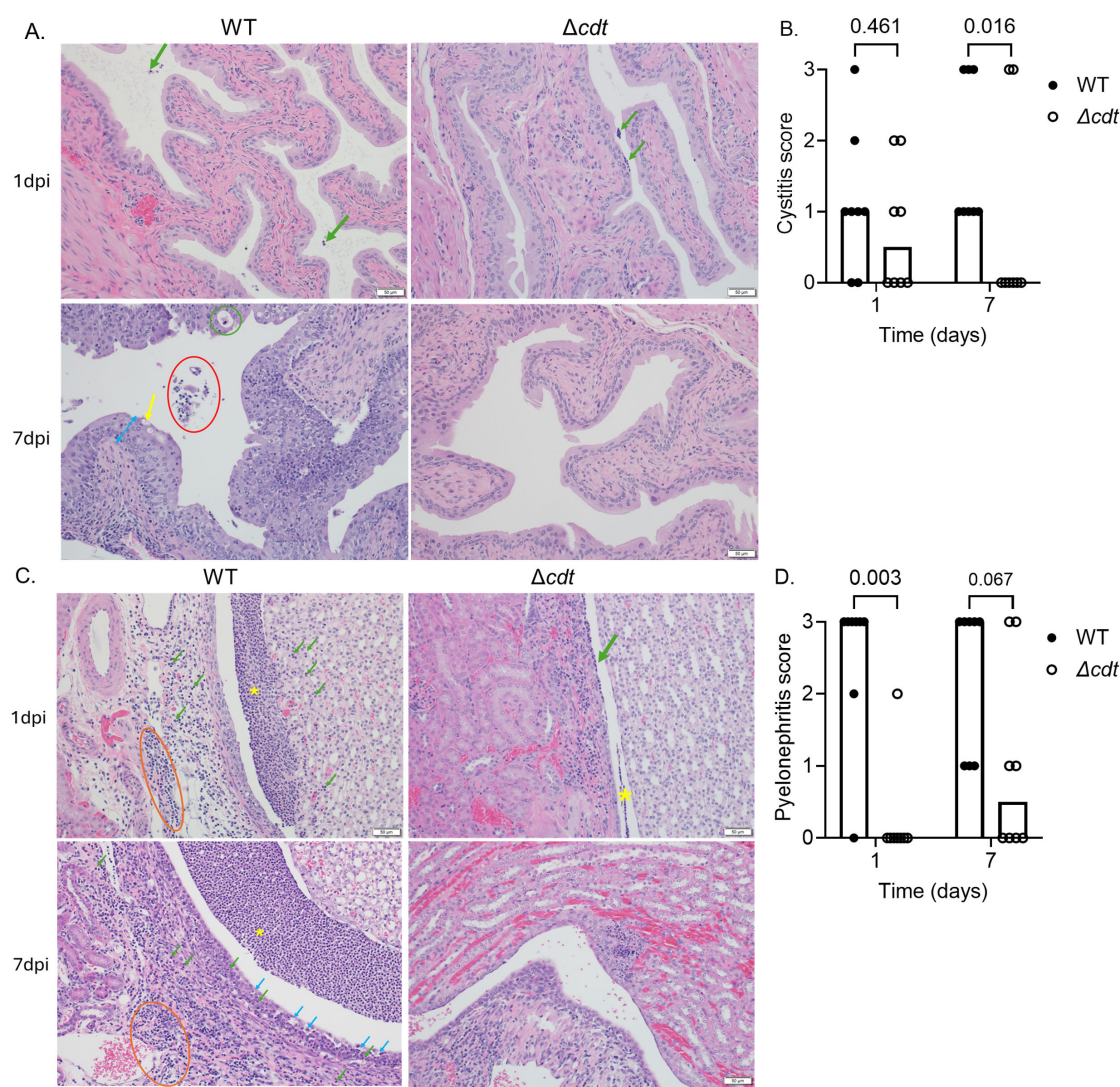
HM56 CDT contributes to cystitis and pyelonephritis pathology during UTI. (**A**) Sections of urinary bladder from mice infected with wild-type (WT) or Δ*cdt* mutant bacteria were stained with hematoxylin and eosin to visualize tissue damage and inflammatory cells. (**B**) The histology scores of individual bladder samples are presented with bars representing the median score. (**C**) Kidney sections from infected mice were stained as described for bladders. (**D**) Histology scores of individual kidneys with bars representing median scores. Images were taken at 200× magnification. Scale bar = 50 µm. Statistical significance in panels B and D was assessed using the Mann-Whitney U test. *P*-values are displayed on the graph, and values < 0.05 were considered statistically significant. Histology key: Green arrow, PMN infiltrates; orange circle, lymphoplasmacytic infiltrates/cuffs; yellow asterisk, PMN rafts; blue arrow, urothelial (mucosal) injury; yellow arrow, vacuolation; green circle, apoptotic body; red circle, sloughed cells and bacteria; and blue double arrow, mucosal hyperplasia.

As a genotoxic effector, CDT has the potential to impact the development of pathology in the urinary bladder and kidneys during UTI. Histological analysis revealed that mice infected with wild-type HM56 developed more severe inflammation and more extensive tissue changes in their kidneys and bladder compared to mice infected with the Δ*cdt* mutant strain (Fig. 5). Specifically, bladders of mice infected with wild-type bacteria displayed more pronounced mucosal hyperplasia with submucosal expansion by edema and infiltrates of inflammatory cells (Fig. 5A). Mucosal injury was also more common and characterized by vacuolated epithelial cells, frequent apoptotic bodies, and sloughed epithelial cells. Inflammation index scoring (Table S1, Fig. S5) demonstrated that 6/8 mice exposed to wild-type HM56 and 4/8 mice exposed to the Δ*cdt* strain showed some level of cystitis at 1 day post-inoculation, with an average severity score of 1.13 and 0.75, respectively (Fig. 5B). At 7 days post-inoculation, the incidence of cystitis was significantly lower in mice infected with the ∆*cdt* mutant (2/8 mice, mean severity = 0.75) compared to mice that received wild-type bacteria (8/8 mice, mean severity = 1.75) (Fig. 5B). Importantly, the bacterial burden of both strains was similar in the bladder after 7 days (Fig. 4C), indicating that the differences in lesion severity between groups were unlikely due to differences in the abundance of bacteria at this site. CDT therefore contributes to HM56-induced pathology of the urinary bladder.

Mice exposed to wild-type HM56 also more frequently developed pyelonephritis or ascending inflammatory lesions in the kidneys centered on the renal pelvis. Pyelonephritis lesions were characterized by prominent rafts of polymorphonuclear cells (PMN), injury of the urothelium with infiltration of PMNs into the renal parenchyma, and expansion of perivascular tissue by edema, PMNs, and lymphoplasmacytic cuffs (Fig. 5C). The inflammation index scoring (Table S1, Fig. S5) showed significantly lower levels of inflammation in the kidneys of mice infected with the Δ*cdt* mutant compared to wild-type at 1 day post-inoculation, and a trend toward lower inflammation at 7 days. Pyelonephritis was observed in 3/8 kidneys after 1 day (mean severity, 0.25) and 2/8 kidneys (mean severity, 1.0) after 7 days in mice inoculated with the Δ*cdt* mutant, compared to 7/8 (mean severity, 2.50) and 8/8 (mean severity, 2.25) at 1 and 7 days, respectively, for kidneys from mice infected with wild-type bacteria (Fig. 5D). These results suggest that CDT contributes to the development and severity of pyelonephritis during UPEC infection.

Genotoxic CDT activity may result in cell death via apoptosis (39). To preliminarily assess this activity in the context of UTI, apoptosis was quantitated by immunohistochemical detection of active caspase-3 (casp3) (Table S2). Casp3 reactivity was not detected in bladders from mice infected with either wild-type HM56 or the Δ*cdt* mutant (Fig. S6A and B). Casp3 reactivity was detected within kidney sections and differed significantly between infection groups. Mice infected with the wild-type HM56 strain showed higher casp3 reactivity (median value of 2.0), with 5/6 mice positive, compared to mice infected with the Δ*cdt* strain (median value of 0), in which only 1/6 mice was positive (Fig. S6A and C). However, it should be noted that reactivity was largely restricted to degenerate PMNs within the rafts in the renal pelvis. Hence, it is not surprising that samples with the greatest severity of pyelonephritis also had the greatest number of casp3-reactive cells. Occasional individual tubular epithelial cells displayed moderate casp3 staining; however, diffuse or regionally extensive tubular involvement was not observed. Together, this suggests that the observed casp3 reactivity is more associated with the progression of inflammatory responses than with the early stages of infection.

## DISCUSSION

CDT is a virulence factor that has been identified in multiple bacterial pathogens and is associated with diverse infection conditions. It has been implicated in the pathogenesis of gastrointestinal disorders including colitis (40) and colorectal cancer (2). For *E. coli*, CDT was initially characterized in gastrointestinal isolates, but it has also been detected in strains recovered from patients with UTI (14, 32). A previous regional study reported that 8% of *E. coli* isolates from UTI cases (*n* = 190) harbor CDT-encoding genes (14). The two strains used here originated from our previous study of uncomplicated UTI isolates and represent 14% of that small cohort (32). A more recent survey of UPEC virulence factors identified the *cdtB* gene in 3% of UTI isolates (*n* = 31) from women ≥ 65 years old compared to 0% (*n* = 15) from asymptomatic bacteriuria cases (33). These data, together with our findings here, support the role of CDT as an accessory virulence factor that contributes to UTI in a subset of UPEC strains. However, broader functional characterization of UPEC CDT variants is warranted, given the observed differences between the HM56 (CDT-IV) and HM57 (CDT-I) strains in this study. Additional explanations for the inconsistent role for CDT in HM56 and HM57 infections include differential CDT abundance between strains, as observed for other UPEC exotoxins (41), or the influence of several other variable virulence factors between UPEC isolates. For example, HM57 is predicted to encode other pro-inflammatory products such as hemolysin (*hly*), cytotoxic necrotizing factor (*cnf*), and colibactin (*clb*) that are not found in the HM56 genome (Table S3). Colibactin is particularly notable for having genotoxic activity (42, 43), which could potentially obscure the loss of CDT in HM57. Distinguishing the contributions of CDT variants within the extensive genotypic diversity of UPEC isolates will require further investigation.

The results from our infection model demonstrate the importance of CDT in the fitness and colonization of UPEC during UTI. In the competitive infections, attenuation of fitness for the ∆*cdt* mutant in the urine, bladder, and kidneys provides compelling evidence that CDT provides a significant advantage for bacterial colonization or survival throughout the urinary tract. In our single-strain infections, bladder and urine UPEC burdens were comparable between wild-type and Δ*cdt* strains, but there was significantly reduced recovery of Δ*cdt* bacteria from the kidneys. Therefore, CDT may either facilitate bacterial ascension to or survival in the upper urinary tract, potentially by modulating the host response or overcoming anatomical barriers. The basis for the fitness defect observed in urine and bladder during co-challenge but not in single-strain infections, despite comparable CFU levels in each, is currently unknown. However, these differences may reflect the increased sensitivity of the co-challenge assays or other additional factors such as nutrient competition, interbacterial antagonism, or differential immune evasion.

Examination of infected bladder and kidney tissues showed that HM56 CDT significantly contributes to urinary tract pathology by enhancing epithelial injury and inflammation. Previous studies have shown that CDT induces DNA damage, leading to cell cycle arrest and apoptosis in epithelial cells, disrupting barrier integrity and facilitating inflammation (24, 44, 45). Epithelial cell damage promotes robust lymphoplasmacytic infiltration and PMN accumulation, as observed here in kidneys and bladders infected with wild-type HM56. These phenotypes were reduced in Δ*cdt* strain, consistent with reports that CDT triggers pro-inflammatory responses and suppresses immune resolution (23, 25, 46, 47). The increased epithelial changes (mucosal hyperplasia, vacuolation, apoptosis, and sloughing) and immune cell infiltration seen here also resemble hallmark pathological features linked to *Helicobacter hepaticus* CDT-mediated genotoxicity in the gastrointestinal tract (46). Our combined results demonstrate that CDT acts as a virulence factor in certain UPEC strains by facilitating bacterial colonization, mediating tissue damage and inflammatory pathology, and exacerbating disease severity in the urinary tract.

## MATERIALS AND METHODS

### Bacterial strains and mutant generation

*E. coli* strains HM56 and HM57 were isolated from uncomplicated UTIs, as previously reported (32), and were routinely cultured at 37°C with aeration in LB (48) at 200 rpm unless stated otherwise. Prophage sequences in bacterial genomes were detected and annotated using PHASTEST (https://phastest.ca), a phage search web-based tool (34). The *cdtABC* locus was deleted (Δ*cdt*) using the bacteriophage lambda-red recombination system as previously described (49). Briefly, primers SP1/SP2 (HM56) and SP5/SP6 (HM57) (Table S4) containing 5′ 35–40 bp sequences homologous to the flanking regions of the *cdt* locus were used to amplify the kanamycin resistance cassette of plasmid pKD4 (35). These PCR products were used to transform HM56 or HM57 harboring pSIM18 (36) by electroporation. Transformants were selected at 30°C on LB agar plates supplemented with kanamycin (50 µg/mL), screened by endpoint PCR using primers SP3/SP4 (HM56) or SP8/SP9 (HM57) (Table S4), and confirmed by Sanger sequencing (Eurofins). Complementation of the HM56 Δ*cdt* mutation was accomplished by ectopic expression on pBBR1MCS-4 (37). The *cdtABC* locus was amplified using primers SP21/SP22 and joined with pBBR1MCS-4 by isothermal assembly with NEBuilder HiFi DNA assembly master mix (New England Biolabs) according to the manufacturer’s recommendations. The recombinant plasmid was confirmed by whole plasmid sequencing (Eurofins) and subsequently introduced, along with the empty vector control, into the deletion mutant by electroporation. Transformants were selected on ampicillin-containing LB medium (100 µg/mL), and the expression of the complemented gene was confirmed by quantitative PCR.

### *E. coli* growth measurements

Overnight cultures of WT and Δ*cdt E. coli* strains in LB were subcultured 1:100 into LB, pooled human urine collected from healthy adult female volunteers, or RPMI 1640 (Gibco), and incubated at 37°C with shaking. Growth was measured by CFU/mL, and population doubling time (DT) was calculated as determined previously (50) from the measured CFUs at a time interval from 1 h to 3 h using the formula: DT = [time duration(log_2_)]/[log(CFU at 3 h) – log(CFU at 1 h)].

To complement CFU kinetics and estimate bacterial growth *in situ*, the O:T^PCR^ method was used as described previously (38). Briefly, cultured bacteria were collected by centrifugation at 4°C and then immediately frozen on dry ice until further processing. Genomic DNA was extracted using the DNeasy Blood and Tissue Kit (Qiagen), and quantitative PCR was performed for gene sequences near the origin (O) and terminus (T) of replication using the primer sequences provided in Table S4. This O:T^PCR^ method was also used to estimate the *E. coli* growth rates in mouse urine during experimentally induced UTI. For this application, urine from mice inoculated with wild-type HM56 and the HM56 Δ*cdt* mutant was collected at 6 and 24 h post-inoculation and then processed as described for the *in vitro* samples.

### Gene expression measurements

HM56 was cultured overnight in LB, then subcultured in LB, and incubated until bacteria reached exponential growth. Bacteria were then harvested, washed in phosphate-buffered saline, and exposed to LB or pooled human urine in three biological replicates for 1 h at 37°C. Bacteria were then stabilized in RNAprotect (Qiagen), collected by centrifugation, and stored at −80°C before RNA isolation. Total RNA was extracted using the RNeasy Kit (Qiagen) and reverse-transcribed with the iScript cDNA Synthesis Kit (Bio-Rad), and quantitative PCR was performed using the primer sequences SP9-12 and PowerUp SYBR Green Master Mix (Applied Biosystems) (Table S4). Both primer pairs were experimentally validated to have amplification efficiencies within 5% of ideal and 5% of each other. The *gyrB* gene was used as the internal control, and relative expression in urine compared to LB was calculated using the 2^-ΔΔCt^ method (51).

### γH2AX immunoblots

T24 bladder carcinoma cells (American Type Culture Collection, HTB-4) were cultured at 37°C with 5% CO_2_ in RPMI 1640 medium supplemented with 10% fetal bovine serum (FBS), penicillin (100 units/mL), and streptomycin (100 µg/mL). *E. coli* HM56 and derivative strains were cultured in LB medium overnight, then harvested by centrifugation, and resuspended in RPMI with 10% FBS. Antibiotic-containing medium was aspirated from T24 cell cultures and replaced with RPMI bacterial suspensions at an MOI = 5 or un-inoculated medium for the positive and negative control conditions. Positive control cells were exposed to a UV-C ultraviolet germicidal lamp for 3 min to induce DNA damage. After 5 h of incubation, the medium was removed from all cultures, and the cells were washed twice with DPBS and then lysed in RIPA buffer (Sigma-Aldrich) containing a protease and phosphatase inhibitor cocktail (Pierce). Lysates were electrophoresed on 4%–20% gradient polyacrylamide stain-free gels (Bio-Rad), imaged, transferred to PVDF membranes, and blocked with bovine serum albumin. The primary antibody consisted of rabbit anti-γH2AX (Abcam, AB81299), and an HRP-conjugated anti-rabbit IgG (Sigma, A6154) was used as the secondary antibody. Blots were developed using SuperSignal West Pico Plus ECL substrate (Thermo). Image analysis was performed with Image Lab (Bio-Rad) software v.6.1. Briefly, the γH2AX signal from each condition was determined relative to cells co-incubated with wild-type HM56 bacteria. Band intensities were then normalized to a subset of proteins observed on the corresponding stain-free image (Fig. S2). The results are representative of three experiments in which T24 cells were either exposed to viable bacteria or bacterial lysates.

### Mouse model of urinary tract infection

All experiments involving animals were performed using protocols approved by the University of Michigan Institutional Animal Care and Use Committee and in accordance with the Office for Laboratory Animal Welfare guidelines. Female 6- to 7-week-old CBA/J mice (Jackson Laboratory) were transurethrally inoculated with *ca*. 10^8^ CFU of *E. coli* as described previously (38). Urine from infected mice was routinely collected at 6 h post-inoculation and daily beginning 24 h post-inoculation. The bladder and kidneys were harvested at 24 h or 7 days to quantify bacterial burdens.

To evaluate the relative fitness of HM56 and the Δ*cdt* mutant derivatives, mice were co-inoculated with a 1:1 mixture of both strains. Urine was collected after 24 h and then the bladder and kidneys were aseptically harvested and homogenized in sterile PBS. Bacterial burdens were determined from urine and tissue homogenates by plating samples on LB agar and LB agar supplemented with kanamycin, which selects for Δ*cdt* mutant bacteria. A CI was calculated using the formula: CI = (mutant CFU recovered/wild-type CFU recovered)/(mutant CFU input/wild-type CFU input). CI were log-transformed and analyzed using one-sample *t*-test against the hypothetical neutral fitness value of 0 to assess statistical significance.

### Histopathology and immunohistochemistry

Histology and immunohistochemistry were performed by the University of Michigan Unit for Laboratory Animal Medicine Pathology Core (RRID:SCR_018823). Urinary bladder and kidneys were harvested at 1 or 7 days post-inoculation from mice inoculated with either wild-type HM56 or the Δ*cdt* mutant strains. Tissues were preserved in 10% neutral-buffered formalin. Bladders were bisected at the approximate level of the trigone, and the kidneys were sectioned transversely, processed, and embedded in paraffin blocks. Sections were cut on a rotary microtome at 4 µm thick, then stained with hematoxylin and eosin for histological evaluation. Representative images were taken using an Olympus DP73 microscope camera and cellSens Entry 4.3 software. The severity and extent of inflammation in each section were scored in a blinded manner by a board-certified veterinary pathologist using the semi-quantitative scoring scheme provided in Table S1. Briefly, the bladder and kidneys were each scored from 0 to 3 for the severity of cystitis or pyelonephritis, with zero signifying no significant lesions and three indicating severe changes. Changes assessed included the type of inflammatory cell infiltrates, the relative numbers of inflammatory cells and their distribution throughout the tissue, and the relative degree of mucosal epithelial cell health. Infiltrates of polymorphonuclear cells (PMN) were weighted more heavily than lymphocytic/lymphoplasmacytic foci. Large rafts or aggregates of PMNs within the pelvis or bladder lumen were weighted more heavily than individualized cells, and samples with inflammatory cells disrupting or extending into the tissue parenchyma were weighted more heavily than those with only intraluminal cells. Mucosal changes included hyperplasia, vacuolation, apoptosis, and sloughing of epithelial cells. In the bladder, the extent of submucosal edema, inflammatory infiltrates, and perivascular cuffs were also included in the score weight. The complete histological scoring criteria, as well as representative images for a given score, are available in the supplementary materials (Table S1, Fig. S5). For immunohistochemistry, the urinary bladder and a kidney from each mouse at 24 h were processed to paraffin, sectioned to glass slides, and stained with cleaved casp3 (Asp175) (Cell Signaling, #9661, 1:1,000) antibody for the evaluation of apoptosis. Reactivity score criteria are provided in Table S2.

## SUPPLEMENTAL MATERIAL

Supplemental material iai.00103-26-s0001.pdf Fig. S1 to S6; Tables S1 to S4.

ASM does not own the copyrights to Supplemental Material that may be linked to, or accessed through, an article. The authors have granted ASM a non-exclusive, world-wide license to publish the Supplemental Material files. Please contact the corresponding author directly for reuse.
