# Supplementary material for: Cytolethal distending toxin enhances Escherichia coli urinary tract infection
Source: Infect Immun. 2026 Jul 13;94(8):e00103-26. doi: 10.1128/iai.00103-26 (PMC13459982; doi:10.1128/iai.00103-26)
Supplement: Supplementary file 1 [file iai.00103-26-s0001.pdf]

Supplementary Figures.

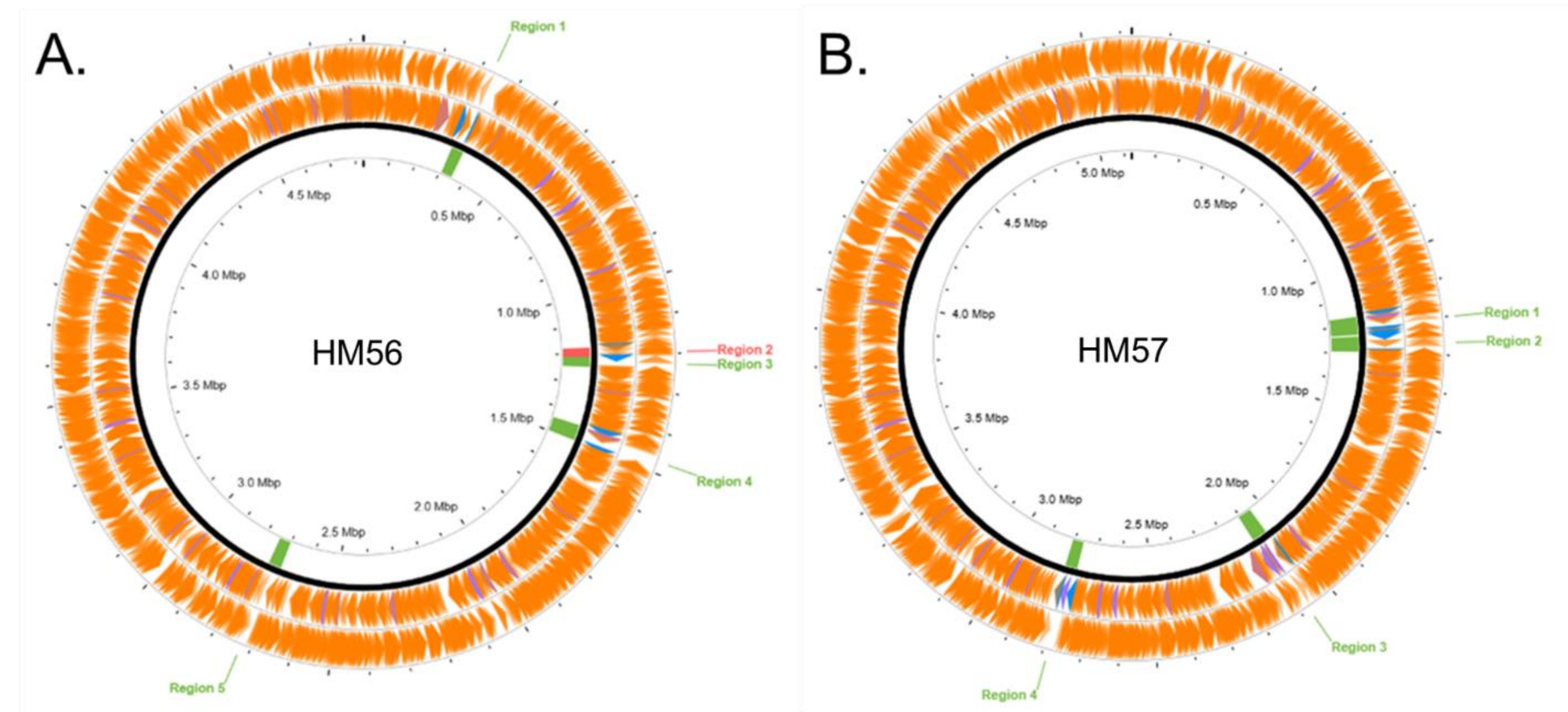

C. <u>UPEC HM56 prophage region summary:</u>

Total:5 prophage regions have been identified, of which 4 regions are intact, 1 regions are incomplete, and 0 regions are questionable.

Region	Region Length	Completeness	Score	# Total Proteins	Region Position	Most Common Phage	GC %	Details
1	42.3Kb	intact	150	55	308390-350735 ⓘ	PHAGE_Shigel_SfII_NC_021857(39)	48.8	Show ⓘ
2	34.5Kb	incomplete	30	18	1177970-1212536 ⓘ	PHAGE_Escher_SH2026Stx1_NC_049919(4)	48.3	Show ⓘ
3	34.1Kb	intact	150	42	1209491-1243682 ⓘ	PHAGE_Entero_DE3_NC_042057(18)	51.9	Show ⓘ
4	54.1Kb	intact	150	61	1443957-1498061 ⓘ	PHAGE_Entero_cdtI_NC_009514(11)	47	Show ⓘ
5	44.1Kb	intact	150	57	2702616-2746777 ⓘ	PHAGE_Salmon_SEN34_NC_028699(24)	49.3	Show ⓘ

Intact (score > 90)
Questionable (score 70-90)
Incomplete (score < 70)

D. <u>UPEC HM57 prophage region summary:</u>

Total:4 prophage regions have been identified, of which 4 regions are intact, 0 regions are incomplete, and 0 regions are questionable.

Region	Region Length	Completeness	Score	# Total Proteins	Region Position	Most Common Phage	GC %	Details
1	65.7Kb	intact	150	67	1167864-1233653 ⓘ	PHAGE_Entero_DE3_NC_042057(17)	50.8	Show ⓘ
2	55.1Kb	intact	150	75	1239271-1294401 ⓘ	PHAGE_Entero_DE3_NC_042057(25)	49.9	Show ⓘ
3	55Kb	intact	150	59	2049351-2104379 ⓘ	PHAGE_Entero_cdtI_NC_009514(12)	47.4	Show ⓘ
4	44.2Kb	intact	130	57	2763977-2808208 ⓘ	PHAGE_Escher_phiV10_NC_007804(40)	48.8	Show ⓘ

Intact (score > 90)
Questionable (score 70-90)
Incomplete (score < 70)

Figure S1. Identification of predicted prophage regions containing *cdt* loci in UPEC strains. A and C. PHASTEST circular genome map and summary table for HM56. The outside orange rings in panel A depict predicted open reading frames. Regions in the inner circle indicate intact (green) or incomplete (red) prophage sequences. Prophage region 4 (1443957-1498061 bp) of HM56 contains the *cdt* gene locus. B and D. PHASTEST prophage predictions for HM57. Prophage region 3 (2049351-2104379 bp) contains the HM57 *cdt* gene locus.

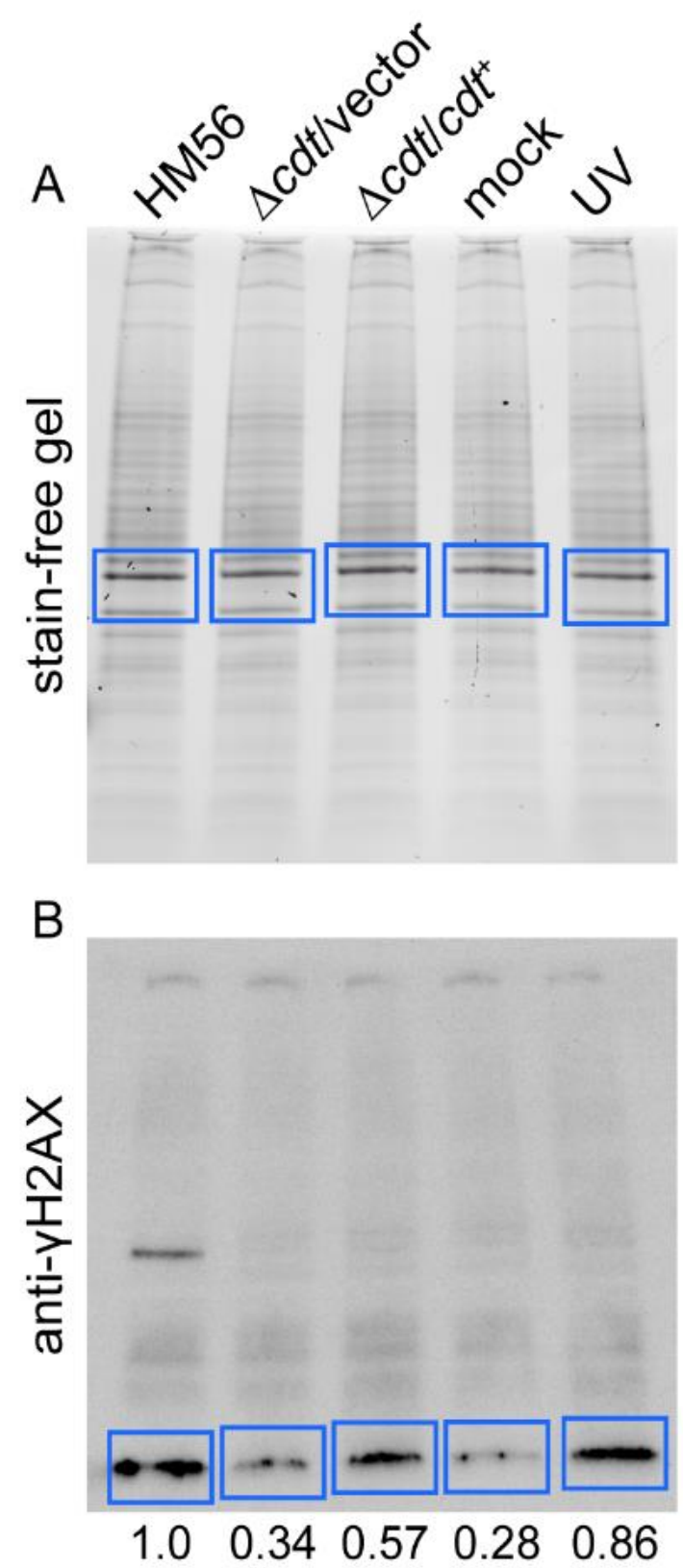

Figure S2. Quantitation of γH2AX in T24 cells exposed to HM56. A. SDS-PAGE of T24 cell lysates used for the γH2AX immunoblot. Total proteins were electrophoresed on a 4-20% gradient polyacrylamide stain-free gel. Blue boxes indicate the approximate regions of each lane that were used for normalization of γH2AX densitometry. B. Complete image of the cropped γH2AX immunoblot shown in Figure 1. Blue boxes outline the approximate regions used for quantitation of γH2AX signal. The number below each lane represents the adjusted γH2AX band volumes relative to HM56 and normalized to cellular proteins from the gel in panel A, as determined using Image Lab software.

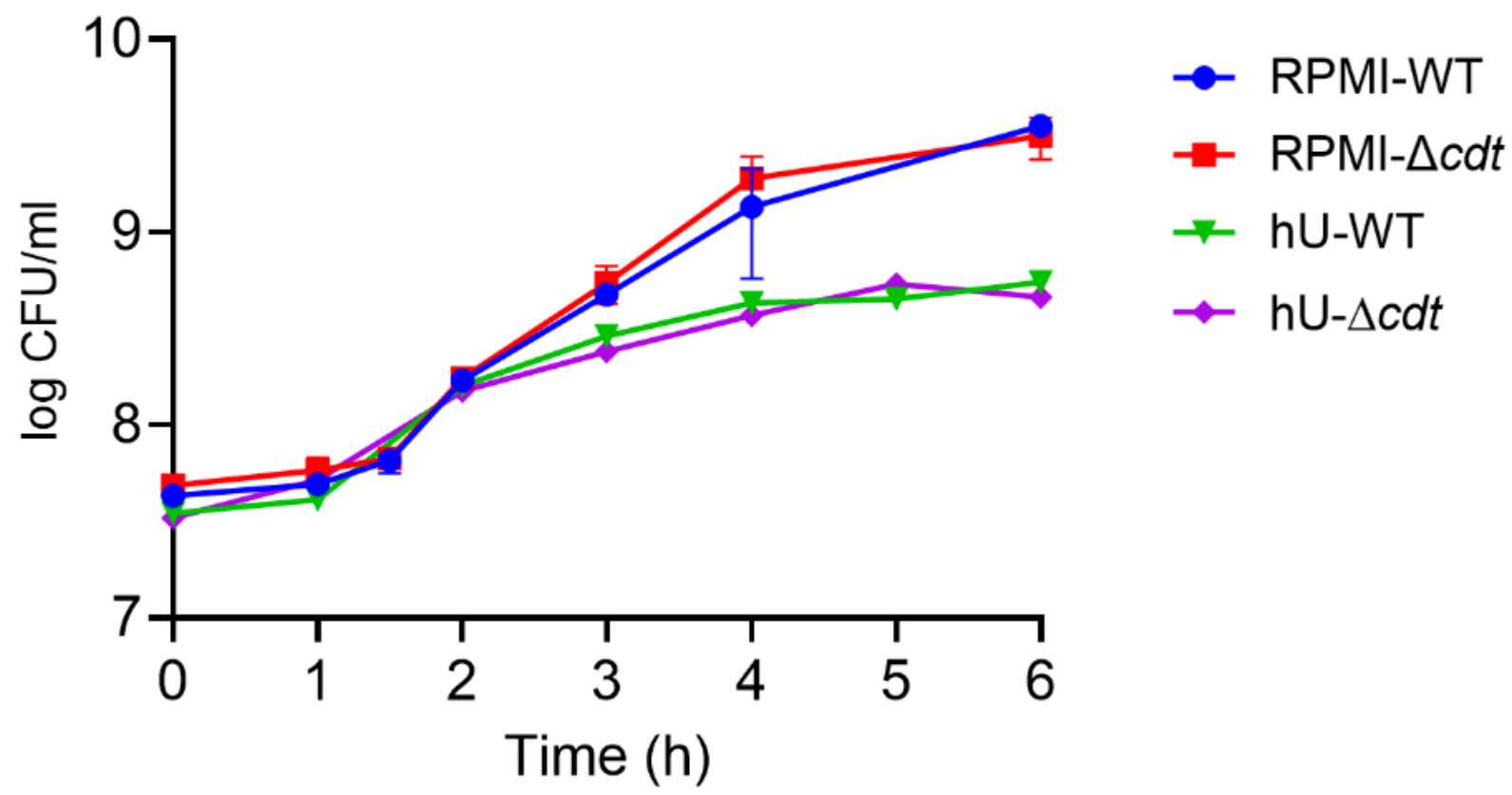

Figure S3. Disruption of *cdt* genes does not influence the growth of HM56 in culture. Wild-type (WT) and Δ*cdt* mutant strains of HM56 were cultured in RPMI cell culture medium (n=2, mean ± standard deviation) or human urine (hU) (n=1).

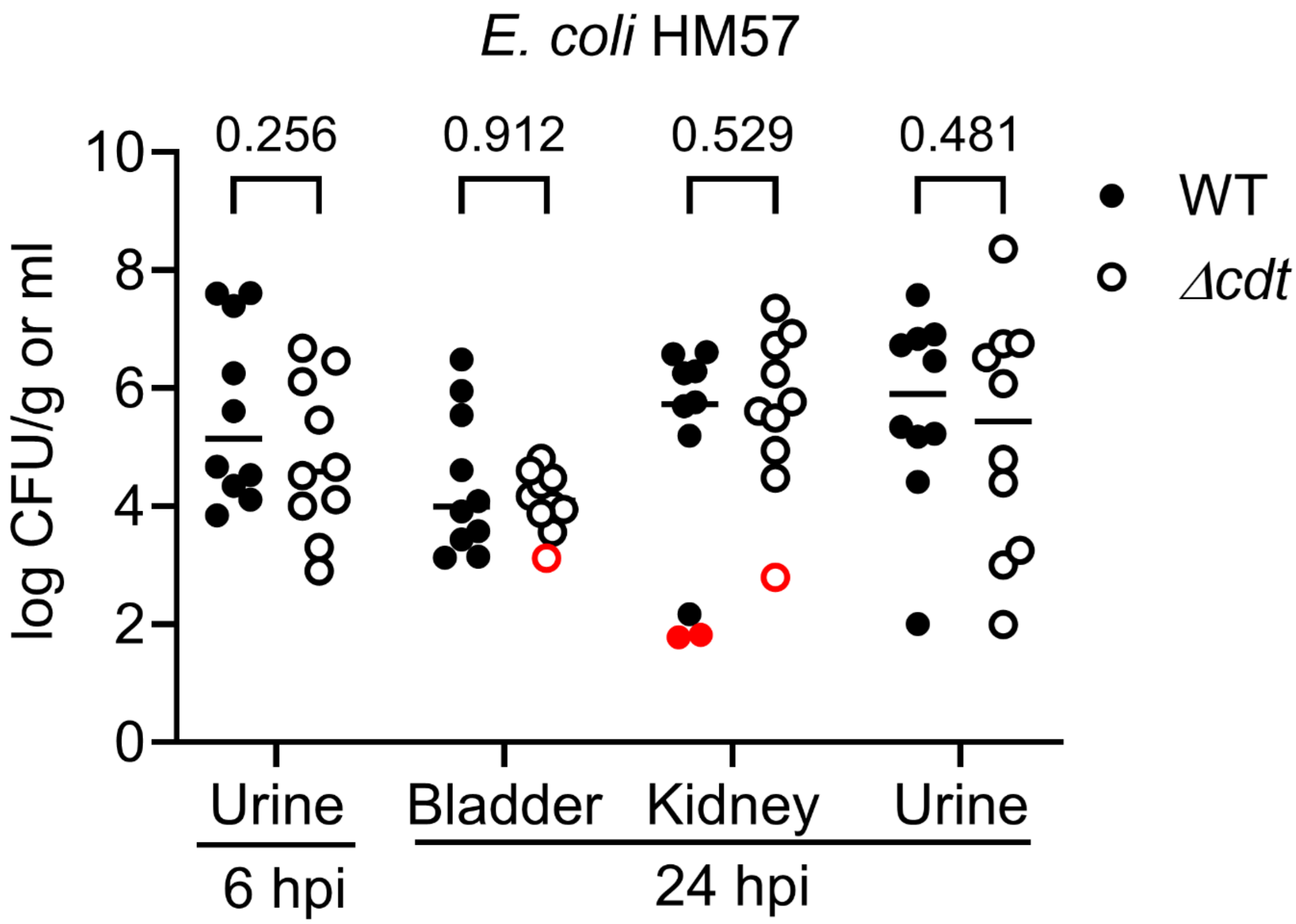

Figure S4: CDT does not significantly contribute to HM57 UTI bacterial burdens. CBA/J mice were transurethrally inoculated with wild-type (WT) HM57 or the Δ*cdt* mutant derivative. Urine was collected at 6 and 24 h post-inoculation, followed by necropsy of the bladder and kidneys for CFU determination. Scatter plots show CFU counts from individual mice (n=10) with the median indicated by lines. Red symbols indicate CFU below the limit of detection. Statistical significance was assessed using the Mann–Whitney U test with P-values displayed above each data pair.

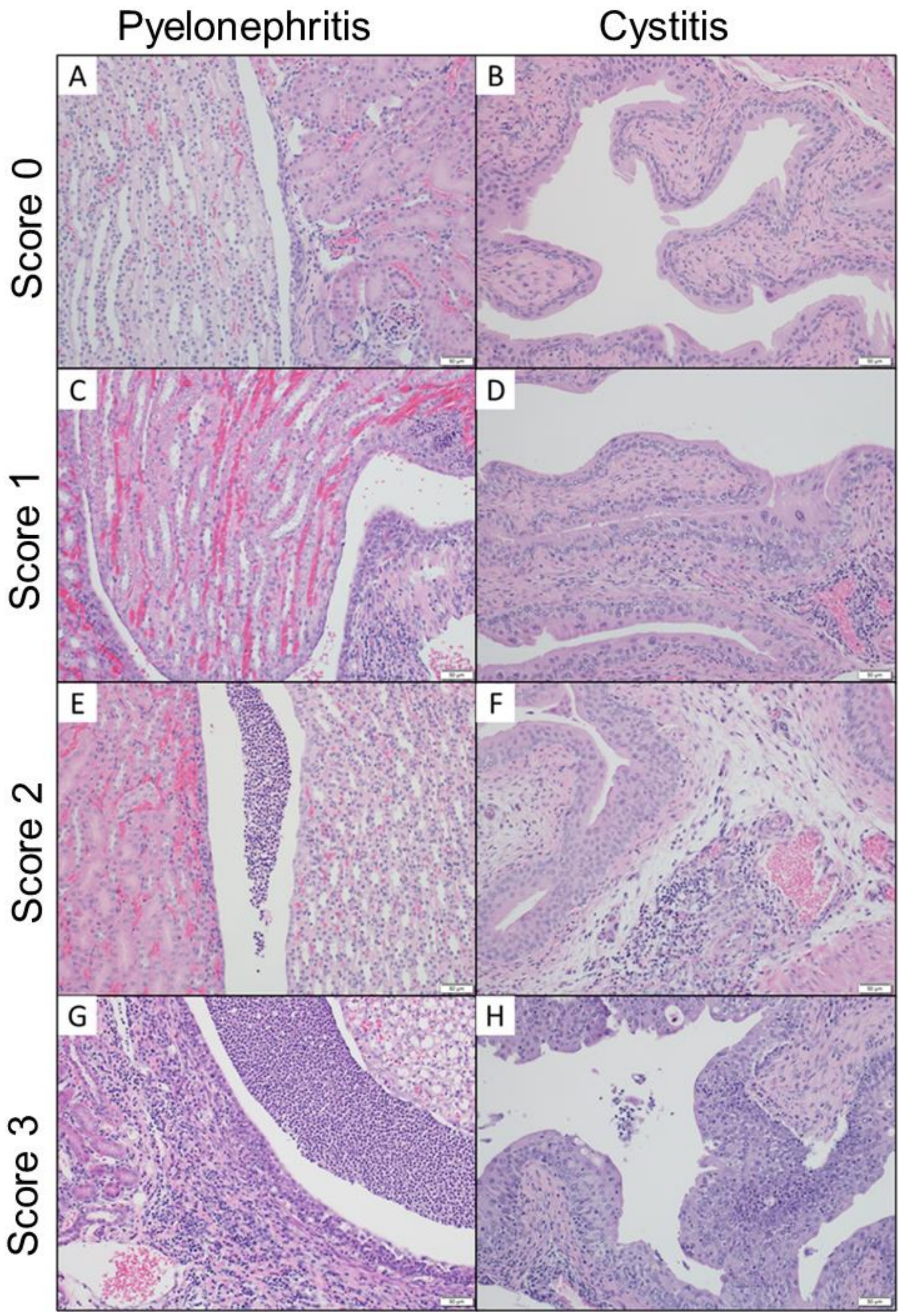

Figure S5. Example tissue sections demonstrating pyelonephritis (left column) and cystitis (right column) severity scores. A and B. Score 0, no significant lesions (NSL). C and D. Score 1, mild lesions. E and F. Score 2, moderate lesions. G and H. Score 3, marked lesions. See Table S3 for definition of NSL, mild, moderate, and marked changes. All sections were stained with hematoxylin and eosin and images were obtained at 200x magnification.

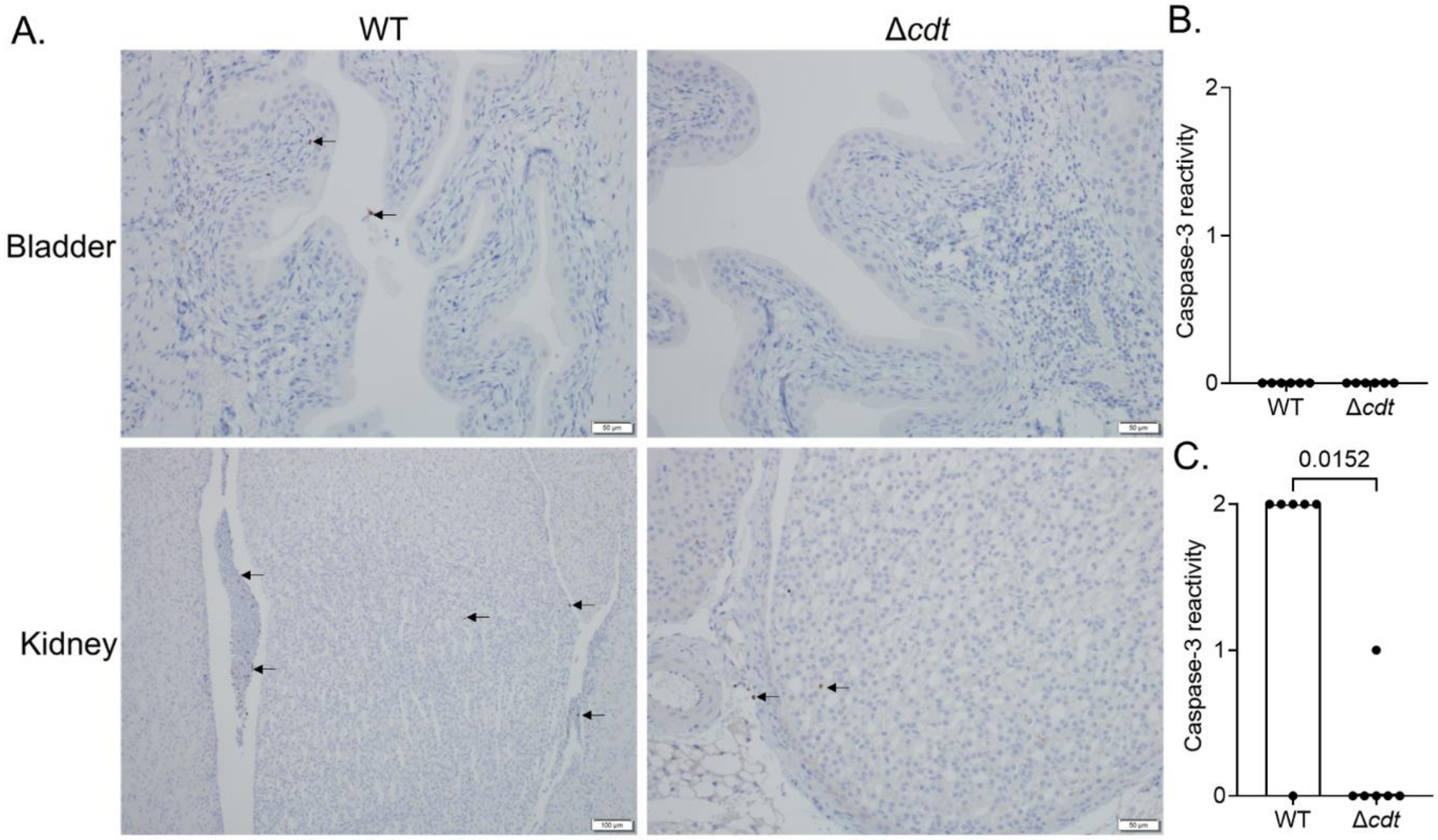

Figure S6. Increased caspase-3 reactivity in kidneys of mice infected with WT HM56. A. Sections of urinary bladder and kidney from mice infected with wild-type HM56 or Δ*cdt* mutant bacteria one day post-inoculation were stained for caspase-3 via immunohistochemistry using a rabbit polyclonal antibody against cleaved caspase-3 (Asp175) (Cell Signaling, #9661) at a 1:1000 dilution. Black arrows indicate regions of brown-staining reactivity. B. The caspase-3 reactivity score of individual bladder samples is summarized with bars representing the median score. C. Caspase-3 reactivity of kidney sections with bars representing the median scores. Statistical analysis was performed using Mann-Whitney U test. Data were considered statistically significant if $P < 0.05$. Images were taken at 200X magnification. Scale bar = 50 μm.

Supplementary Tables.

Table S1. Histopathology scoring criteria.

	**Score**			
	**0 - NSL**	**1 - Mild**	**2 - Moderate**	**3 - Marked**
**Pyelonephritis**	No Significant Lesions (NSL) There is a lack of inflammatory infiltrates in either the renal pelvis or parenchyma, no evidence of mucosal injury or reactive changes, and no interstitial changes (e.g. edema or fibrosis).	Very occasional infiltrates of PMNs or small foci of lymphoplasmacytic cells in either the renal pelvis or superficially extending into the renal parenchyma. No mucosal change. May see lympho-plasmacytic vascular cuffs.	PMN inflammatory infiltrates are more common. May see small rafts of PMN aggregates in the renal pelvis or small scattered foci of PMNs infiltrating into the superficial renal parenchyma. Minimal mucosal change.	Many PMN infiltrates are present, forming large rafts which distend the renal pelvis. Infiltration of PMNs into the renal parenchyma may extend deeper, and mucosal changes are more common (vacuolation, degeneration, hyperplasia).
**Cystitis**	NSL There is a lack of inflammatory infiltrates in either the wall or lumen of the bladder . There are no changes to the mucosa or submucosal tissues.	Rare PMN infiltrates within the bladder lumen (individualized cells), or small perivascular infiltrates – Lymphocytes and plasma cells predominate over PMNs. No mucosal changes (hyperplasia / injury), and no expansion of the submucosa (edema / inflammation).	Increased numbers of PMNs cells, commonly observed. Infiltrates may be observed in the lumen, mucosa, or submucosa. May see mild to moderate mucosal changes, and there may be mild or focal submucosal edema.	Many PMN infiltrates are present, observable in most microscopic fields, and may be transmural. Mucosal changes are extensive – Commonly see areas of mucosal injury (vacuolated, apoptotic, or sloughed urothelial cells and erosion). Hyperplasia is often marked and diffuse.

Table S2. Caspase-3 immunohistochemistry scoring criteria.

Score	Caspase-3 reactivity
-	Only reactive cells are rare (<5) positive tubular cells. Within the degree of normal expression.
+	Reactive cells are largely isolated to degenerate neutrophils within rafts of inflammatory cells.
++	Rafts of inflammatory cells within the pelvis and foci of inflammation within the adjacent parenchyma are commonly immunoreactive (~5-10% of cells). Low numbers of epithelial cells within the parenchyma adjacent to the pelvis are also immunoreactive.

Table S3. Predicted protein toxins and metabolites encoded in UPEC strains HM56 and HM57.

Toxins	HM56	HM57
cytolethal distending toxin (*cdt*)	+	+
hemolysin (*hly*)	-	+
cytotoxic necrotizing factor (*cnf*)	-	+
serine protease autotransporter (*vat*)	+	+
colibactin (*clb*)	-	+

+, present

-, absent

Table S4. Oligonucleotides used in this study.

ID	Primer	Sequence (5′-3′)	Reference
SP1	HM56_*cdtABC*_KO-F	ctaattgcatttttgtgcacacttataattactggttgctGTGTAGGCTGGAGCTGCTTC	This study
SP2	HM56_*cdtABC*_KO-R	ccatttagaattgcaaggcattcattaccatcagggttttATATGAATATCCTCCTTAGT	This study
SP3	HM56_*cdtABC*_KO_confirm-F	CTCCATTCCCCAAACATTGACCA	This study
SP4	HM56_*cdtABC*_KO_confirm-R	GTATTACGTAATGAAACAGACTGGCG	This study
SP5	HM57_*cdtABC*_KO-F	tataaaaaagaggtggtgcagaggaggaaatacagtgGTGTAGGCTGGAGCTGCTTC	This study
SP6	HM57_*cdtABC*_KO-R	tcagctcgttaatggagacattattgccggagataatggtATATGAATATCCTCCTTAGT	This study
SP7	HM57_*cdtABC*_KO_confirm-F	GCGAGTATCTACCGAACCGTA	This study
SP8	HM57_*cdtABC*_KO_confirm-R	CCGTTTTGCCTGTGTGTTGA	This study
SP9	*E. coli gyrB*-F	AGTACA ACCCGGACA AACTG	This study
SP10	*E. coli gyrB*-R	GGCATCTGACGATAGAAGAAGG	This study
SP11	HM56 *cdtB*-F	GCCGGGTATTTCTTTTGCAGA	This study
SP12	HM56 *cdtB*-R	TATCAACAGCACCACTTCCC	This study
SP13	*E. coli*_*oriC*-F	CGCAACAGCATGGCGATAAC	(35)
SP14	*E. coli*_*oriC*-R	TTCGATCACCCCTGCGTACA	(35)
SP15	*E. coli*_*terC*-F	TCAACGTGCGAGCGATGAAT	(35)
SP16	*E. coli*_*terC*-R	TTGAGCTGCGCTTCATCGAG	(35)
SP17	UPEC_*oriC*-Probe	/56-FAM/ACCCATGAT/ZEN/GTGATCCGCAGTAACCTCGATCGTA/3IABkFQ/	(35)
SP18	UPEC_*terC*-Probe	/5SUN/CGCGCTAAA/ZEN/CCCGCCCTGCTGCTTAT/3IABkFQ/	(35)
SP19	pBBR1-F	CGTAATCATGGTCATAGCTGTTTCC	This study
SP20	pBBR1-R	TGAGTCGTATTACGCGCGCTCACTG	This study
SP21	pBBR1-MCS4_*cdt*-F	CAGTGAGCGCGCGTAATACGACTCAAGTCATCATATCAGGGGAAC	This study
SP22	pBBR1-MCS4_*cdt*-R	GGAAACAGCTATGACCATGATTACGTTATCTGAACATGCCCCCCG	This study
SP23	pBBR1_*cdt*_confirm-F	GTAAAACGACGGCCAG	This study
SP24	pBBR1_*cdt*_confirm-R	CAGGAAACAGCTATGAC	This study
